# Modified conjoint fascial sheath suspension for the correction of severe congenital blepharoptosis in pediatric patients at different ages

**DOI:** 10.3389/fped.2022.954365

**Published:** 2022-10-21

**Authors:** Huixing Wang, Zhaochuan Liu, Yadi Li, Lihua Song, Runhui Pang, Jianwei Yang, Ping Bai

**Affiliations:** ^1^ Department of Ocular Plastic, Hebei Eye Hospital, Hebei Provincial Key Laboratory of Ophthalmology, Hebei Provincial Clinical Research Center for Eye Disease, Xingtai, China; ^2^ Department of Ophthalmology, Beijing Tongren Hospital, Beijing Ophthalmology and Visual Science Key Lab, Capital Medical University, Beijing, China; ^3^ Department of Ophthalmology, Affiliated Hospital of Yunnan University, Key Laboratory of Yunnan Province, Yunnan Eye Institute, Kunming, China

**Keywords:** general anesthesia, pediatric blepharoptosis, conjoint fascial sheath (CFS), severe congenital blepharoptosis, levator muscle

## Abstract

**Objective:**

To evaluate the surgical outcomes of modified combined fascia sheath (CFS) and levator muscle (LM) complex suspension for the correction of severe congenital blepharoptosis in pediatric patients.

**Methods:**

Pediatric patients with severe congenital blepharoptosis were enrolled form July 2017 to July 2021. All patients were divided into two groups according to their age (group A ≤ 7 years; group B > 7 years) and received CFS + LM suspension surgery. Main surgical outcome indexes include margin reflex distance 1 (MRD1) and MRD1 regression. Postoperative complications such as lagophthalmos (LAG), conjunctival prolapse, exposure keratopathy and trichiasis were documented.

**Results:**

Fifty patients (60 eyes) were enrolled, including 17 patients (18 eyes) in group A and 33 patients (42 eyes) in group B. The MRD1 in group A was 3.06 ± 0.64 mm at 6 months after the operation, and the MRD1 in group B was 2.64 ± 0.69 mm 6 months postoperatively which is significantly lower than that of group A (*P *= 0. 044). At the last visit, however, the MRD1 in group A was 3.00 ± 0.69 mm and the MRD1 in group B was 2.64 ± 0.70 mm. There was no significant difference in MRD1 between two groups in long term (*P *= 0.255). Additionally, there were a variety of degrees of MRD1 regression, especially in the first month after the operation in both groups (both *P *< 0.001). Moreover, there were 9 cases of postoperative complications in group A and 13 cases in group B. The overall occurrence of postoperative complications in group A was significantly lower than that in groups B (*χ*^2^* *= 4.413, *P *= 0.036).

**Conclusions:**

CFS + LM suspension, a modified CFS-based surgery, is an effective treatment for severe congenital blepharoptosis in pediatric patients. Moreover, CFS + LM suspension demonstrate excellent long-term outcomes, including good movement of the eyelid, satisfied eyelid closure and fewer postoperative complications.

## Key messages

1. Congenital blepharoptosis is very common in clinical practice, and surgery is the only effective treatment at present.
2. Modified conjoint fascial sheath suspension has shown good results for the treatment of severe blepharoptosis.
3. The purpose of this study was to analyze the applicability of this technique in pediatric patients.
4. The operations, which were performed under general anesthesia, were found to be more challenging.

## Introduction

Congenital blepharoptosis is mostly caused by dysplasia of the oculomotor nucleus or levator muscle (LM), and patients mainly show partial or complete blepharoptosis. It has been estimated that levator function is poor in 71.8% of eyes with congenital blepharoptosis, and unilateral cases account for 64.7%–75.0% (1, 2). Patients with congenital blepharoptosis, especially unilateral cases, are more susceptible to the development of amblyopia, usually due to convergent strabismus, high astigmatism, or anisometropia (3). Blepharoptosis can be cosmetically, functionally, and psychosocially problematic for children. This disease can be divided into 3 grades: mild, moderate, and severe. In mild cases, the upper eyelid margin is located on the upper edge of the pupil, and the amount of blepharoptosis is 1–2 mm; in moderate cases, the upper eyelid margin covers 1/3 of the pupil, and the amount of blepharoptosis is 3–4 mm; in severe cases, the upper eyelid margin covers 1/2 of the pupil, and the amount of blepharoptosis is >4 mm (4).

In the past, frontalis muscle flap suspension was used for the surgical treatment of severe blepharoptosis (5). Postoperative complications such as poor ocular mobility, subcutaneous hematoma and obvious lagophthalmos are often troublesome for surgeons. Since 2002, conjoint fascial sheath suspension (CFS) has been applied to correct congenital blepharoptosis (6). This procedure has a satisfactory curative effect in mild or moderate blepharoptosis, however, the effect of the surgery on severe ptosis was not ideal (6, 7). Over the past few years, CFS + LM complex suspension, a modified CFS-based surgery, has shown good results for the treatment of severe blepharoptosis (7–9). Many anatomical and histological studies have also provided helpful information (9, 10). Recent research has confirmed that the CFS is rich in collagen fibers and elastic fibers (11). Additionally, our previous study confirmed that the CFS and LM are rich in elastic fibers and elastin in pediatric patients, and CFS-based suspension is effective to cure severe congenital blepharoptosis in pediatric patients (9). In the present study, we performed CFS + LM suspension to treat severe congenital blepharoptosis in pediatric patients in different age groups and evaluated the efficacy and safety of this procedure.

## Materials and methods

### Patients

Pediatric patients with severe congenital blepharoptosis from July 2017 to July 2021 were included in this study. Patients were divided into group A (≤7 years old) and group B (>7 years old). All patients underwent CFS + LM suspension, a modified CFS-based surgery, under general anesthesia performed by one experienced surgeon (P.B.). The inclusion criteria included patients older than 3 years old and younger than 15 years old; clinically diagnosed severe congenital blepharoptosis; normal ocular movement on preoperative examination; no serious autoimmune diseases or connective tissue diseases; no other contraindications for eyelid surgery; capability of attending follow-up visits for at least 6 months after treatment. The exclusion criteria included patients with contraindications for eyelid surgical intervention, patients with oculomotor nerve palsy, patients with immune diseases or connective tissue diseases, patients with systemic diseases, and patients with psychosis history. This study was consistent with the Declaration of Helsinki, and it was approved by the Ethics Committee of Hebei Provincial Eye Hospital. Written informed consent forms were obtained from the parents of all patients.

### CFS + LM suspension technique

**Figure 1:**
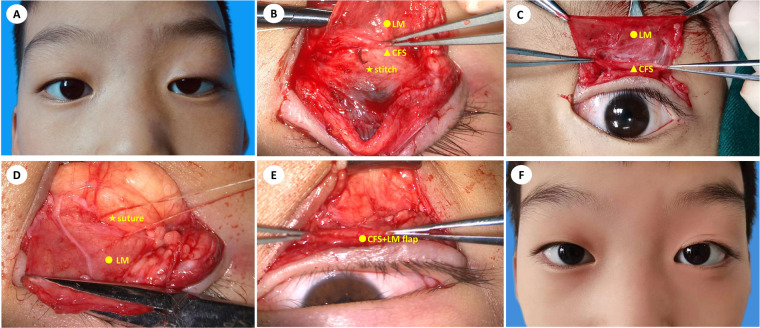
An 8-year-old boy with congenital ptosis (OS) treated by modified CFS suspension under general anesthesia. (**A**) preoperative image; (**B**) suturing CFS and LM with 5-0 absorbable stitches; (**C**) exposure of CFS and LM; (**D**) using mattress sutures to fix CFS + LM complex flap onto the tarsus; (**E**) exposure of the CFS + LM complex flap; (**F**) twelve months after CFS + LM suspension.

All CFS + LM suspension surgeries were under general anesthesia performed by the same surgeon (P.B.) using the same technique (9). The surgical incision consisted of a double eyelid line incision and its course was marked with methylene blue preoperatively. The upper lid was anesthetized using ropivacaine plus 1:10,000 epinephrine. Initially, the skin was cut along the marked line, and the subcutaneous tissue was separated. Then, the orbicularis oculi was cut at a width of 2 mm before the tarsal plate. The aponeurosis of the levator was cut to the same length as the incision parallel to the margin of the tarsal plate. The levator aponeurosis was carefully separated from the Müller muscle, leaving the Müller muscle and conjunctiva intact. The levator aponeurosis was detached from the Müller muscle and pulled approximately 4–5 mm over the fornix to expose the CFS. As shown in Figure 1, the CFS is a sheath-like connective tissue whose thickness varies among individuals. The upper margin of the tarsus was suspended and fixed to the CFS + LM complex at a certain length according to patients' margin reflex distance 1 (MRD1). With reference to the position of the tarsus and pupil, U-shaped stitches of 5-0 absorbable thread were applied at the outer, intermediate, and inner positions. Then adjust the upper eyelid height to a satisfactory position, and the skin incision was closed with 5-0 nylon stitches, which were removed one week after surgery.

### Postoperative evaluation

On the second day after surgery, the bandage was removed, and the skin incision was wiped with iodophor daily. Sodium hyaluronate eye drops were given 4 times a day, and erythromycin eye ointment was administered once every night. Eyelid sutures were removed on the seventh day after surgery. The follow-up time points were preoperative, 1 week after the operation, 1 month after the operation, 3 months after the operation, 6 months after the operation, and the last follow-up. The MRD1, lagophthalmos (LAG), and complications after the operation were recorded and analyzed by experienced ophthalmologists (R.P. and J.Y.). MRD1 was determined by the ophthalmologist and patient aligning at the same level. During the examination, the patient was instructed to gaze in the primary position, after which a light is directed at the patient's eyes and the distance between the light reflex on the patient's cornea to the upper-eyelid margin was measured in millimeters with a ruler. LAG is the inability to close the eyelids completely on attempted closure. During the test, the patient was instructed to look down and gently close both eyes, and the distance between the upper and lower eyelid margins was measured with a ruler in millimeters. In terms of cosmetic evaluation, the symmetry of lid height, lid contour, and lid crease were recorded.

The upper eyelid located 1–2 mm below the upper corneal margin was considered to be well corrected; the upper eyelid located at or above the upper corneal margin was considered to be overcorrected; and the upper eyelid located >2 mm below the upper corneal margin was considered to be undercorrected (12).

### Statistical analysis

Statistical analysis was performed using SPSS 20.0 software (SPSS Inc., Chicago, IL, USA). A normality test was performed for each continuous variable, and two independent sample *t* tests were performed for normally distributed data, while *rank sum* tests were performed for nonnormally distributed data. The *chi-square* test was used for the comparison of categorical variables between groups. Measurement values are presented as the mean ± standard deviation. A *P*-value <0.05 was considered statistically significant. GraphPad Prism 8.0 was used for drawing.

## Results

Fifty pediatric patients (60 eyes) undergoing modified CFS suspension for severe congenital blepharoptosis were included in the study. The demographic characteristics of the study population were summarized in Table 1. There were 17 patients (18 eyes) in group A (12 males and 5 females), with an average age of 5.76 ± 1.15 years and an average preoperative MRD1 of −1.17 ± 1.20 mm. The average LF was 1.50 ± 1.29 mm. There were 33 patients (42 eyes) in group B (26 males and 7 females), with an average age of 10.09 ± 1.67 years and an average preoperative MRD1 of −0.99 ± 0.77 mm. The average LF was 1.98 ± 1.39 mm. All patients had normal ocular movement on preoperative examination and were followed up for at least 6 months after surgery. There was no difference in general data between the two groups (*P* > 0.05).

**Table 1:** Preoperative patient data.

Characteristics	Group A	Group B	*P*-value
*n*	17	33	
Sex, *n*			0.769^a^
Female	5	7	
Male	12	26	
Laterality, *n*			0.156^a^
Unilateral	16	24	
Bilateral	1	9	
Age (years)	5.76 ± 1.15	10.09 ± 1.67	
Preoperative			
MRD1, mm	−1.17 ± 1.20	0.76 ± 1.16	0.166^b^
LF, mm	1.50 ± 1.29	1.98 ± 1.39	0.289^b^

MRD1, margin-to-reflex distance; LF, levator function.

^a^ *χ*^2^.

^b^ Rank sum test.

### Corrective effects

**Figure 2:**
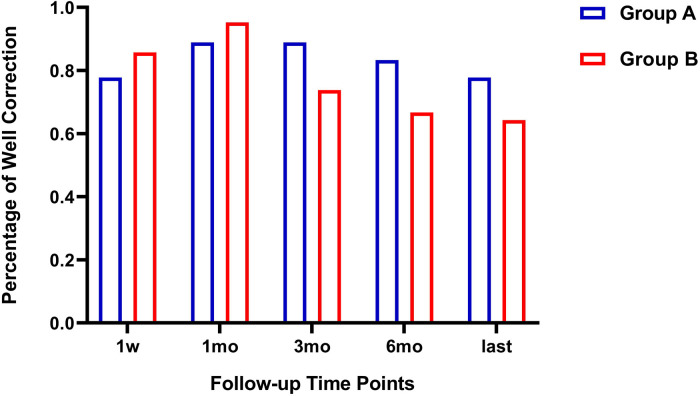
Comparison of corrective effect between two groups at different follow-up points.

In group A, 4 eyes were overcorrected within 1 week after the operation; 14 eyes were well corrected and 4 eyes were undercorrected after the operation at the last visit. In group B, 4 eyes were overcorrected and 2 eyes were undercorrected within 1 week after the operation; 27 eyes were well corrected and 15 eyes were undercorrected after the operation at the last visit. The correction rate was not significantly different between group A and group B at the last follow-up visit (Figure 2).

### Postoperative MRD1

**Figure 3:**
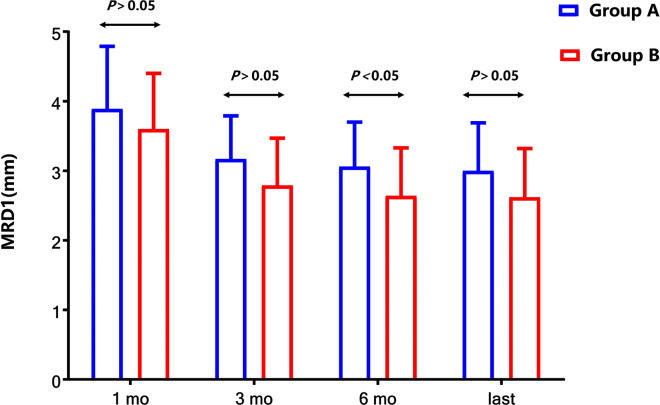
Comparison of MRD1 between two groups after surgery. Compared with the MRD1 values in group B, the values of MRD1 at 6 months after the operation were elevated significantly (*P *< 0.05), but those at the last follow-up were not significantly elevated (*P* > 0.05).

The MRD1 results in group A and group B at different follow-up points were listed in Table 2. The MRD1 in group A was 3.06 ± 0.64 mm at 6 months after the operation, and the MRD1 in group B was 2.64 ± 0.69 mm 6 months postoperatively which is significantly lower than that of group A (*P *= 0.044). As is shown in Figure 3, however, the MRD1 in group A at the last visit was 3.00 ± 0.69 mm and the MRD1 in group B was 2.64 ± 0.70 mm. There was no significant difference in MRD1 between two groups in long term (*P *= 0.255).

**Table 2:** MRD1 results (mm) in two groups at different follow-up points.

	preoperative	1 week	1 month	3 months	6 months	Last follow-up
Group A	−1.17 ± 1.20	4.89 ± 0.76	3.89 ± 0.90	3.17 ± 0.62	3.06 ± 0.64	3.00 ± 0.69
Group B	−0.76 ± 1.16	4.52 ± 0.99	3.60 ± 0.80	2.79 ± 0.68	2.64 ± 0.69	2.62 ± 0.70
*P*	0.166	0.256	0.248	0.050	0.044	0.255

### Upper eyelid regression

**Figure 4:**
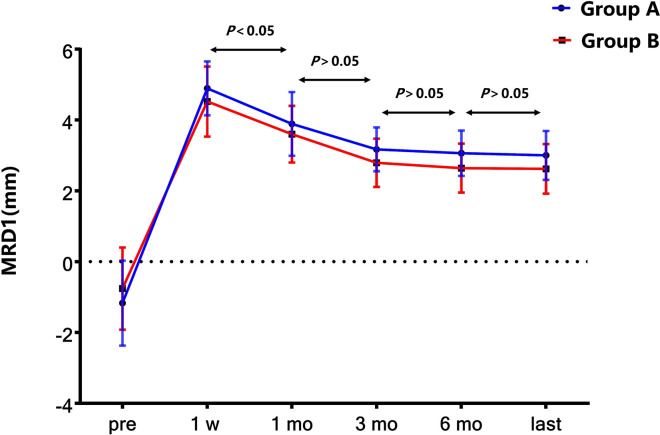
MRD1 regression in two groups at different follow-up points.

The intraoperative palpebral fissure height (PFH) was set at 8 mm, and the measurement of MRD1 was made in each patient within 1 week after the operation. However, the upper eyelid regressed gradually in group A and group B. MRD1 regression was most evident during the first month and gradually stabilized after the third month in both group A and group B (Figure 4). It was suggested that the first month after the operation was the key period of this treatment.

### Postoperative complications

All patients had early incomplete eyelid closure, which resolved at 1 month after surgery. Two patients presented exposure keratopathy in the follow-up period. The number of eyes with lagophthalmos greater than 2 mm after the operation at the last visit was recorded. Nine patients (9 eyes) showed conjunctival prolapse within 1 week after surgery, and one patient showed conjunctival prolapse 10 days after surgery among these 50 patients (60 eyes), including 4 patients (4 eyes) in group A and 6 patients (6 eyes) in group B. Among them, 7 patients recovered after fixation of the conjunctival fornix with deep sutures, and the remaining 3 patients with mild conjunctival prolapse recovered after conservative treatment, such as eye drops and bandaging. There were fewer postoperative complications in group B than in group A (*P *< 0.05), as shown in Table 3.

**Table 3:** Comparison of postoperative complications between two groups.

Complication	Group A	Group B	*χ* ^2^	*P*
Lagophthalmos greater than 2 mm	4 (4/18)	6 (6/42)		
Conjunctival prolapse	4 (4/18)	6 (6/42)		
Exposure keratopathy	1 (1/18)	1 (1/42)		
Trichiasis	0 (0/18)	0 (0/42)		
Total incidence rate	9 (9/18)	13 (13/42)	4.413	0.036

## Discussion

Congenital blepharoptosis is very common in clinical practice, and surgery is the only effective treatment at present. Pediatric blepharoptosis is more challenging than adult blepharoptosis because of extra considerations such as the risk of amblyopia, difficulty of examination, need for general anesthesia, and the age at which the surgery should be performed (13). LF and severity of blepharoptosis determine the choice of surgical treatment for congenital blepharoptosis (14). For severe blepharoptosis with poor LF, frontalis suspension is generally considered to be the most effective method and is especially recommended for patients with unilateral congenital blepharoptosis (15). In previous studies, frontalis suspension was used to treat unilateral severe blepharoptosis with a success rate of 77.0%–95.0% (16). Many different materials used in frontalis suspension surgery are readily available and can shorten operative times, but they can also lead to the recurrence of blepharoptosis or complications related to allogeneic materials such as infection, graft exposure, rejection and granuloma formation. Lid lag on downward gaze after frontalis suspension has also been reported in up to 90% of cases (17). Unilateral frontalis suspension may show less satisfactory outcomes, which result from ipsilateral nondominant eye amblyopia and failure to elevate the forehead (18). Mild-to-moderate exposure keratopathy can develop in all patients after the correction of severe congenital blepharoptosis, regardless of the procedure (19). Entropion or eyelash inversion occurs in 5.4%–11.9% of cases after correction of severe congenital blepharoptosis, which is associated with increased posterior lamella vertical tension (16, 20). In recent years, CFS suspension has been used to correct severe blepharoptosis, and the therapy has been widely recognized by clinicians. CFS suspension has achieved good results in the treatment of severe blepharoptosis (7). CFS is the fusion of fascia between the levator muscle and the superior rectus muscle, and its efficacy derives from the combined strength of the muscle belly of the levator muscle and the Tenon capsule. Numerous histological studies of CFS were published many years ago. The previous reports on the efficacy of CFS suspension mainly focused on adults (21), while congenital blepharoptosis was found to be more common in children. In this study, 50 patients (60 eyes) ranging from 4 years to 14 years with severe congenital blepharoptosis were recruited to analyze and compare the applicability of this technique in pediatric patients at different ages.

In 2002, Holmström and Santanelli found abundant collagen fibers and elastic fibers in a histological study of CFS (10) Our previous study confirmed that CFS contains abundant collagen fibers and elastic fibers (9). The appearance of CFS tissue in children is yellowish and whitish with varying thickness, which is not obviously different in appearance and elasticity compared with those in adults. Traditional CFS suspension has a high rate of upper eyelid retraction and blepharoptosis recurrence (7). The CFS + LM suspension can strengthen the longitudinal and upward traction force, and maintains the original physiological structure of the upper eyelid. It is thus easier to achieve a natural and beautiful radian of the eyelid margin in children, and to avoid deformities of the eyelid margin in the long term. Motility is obviously more consistent with the physiological structure, and good blinking function can be maintained to a certain extent. Meanwhile, CFS + LM suspension also prevents upper eyelid hypertrophy and scarring and does not depend on eyebrow elevation when opening the eyes, unlike frontalis suspension.

**Figure 5:**
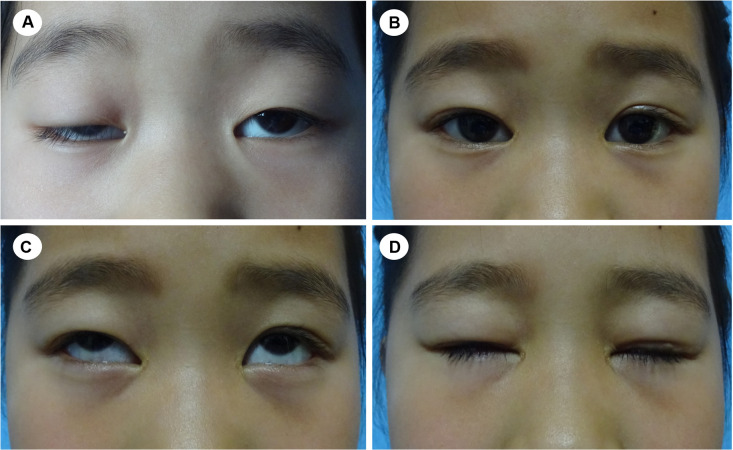
A 6-year-old girl with congenital blepharoptosis OU. (**A**) preoperative appearance; (**B**) image of the patient eight months after CFS + LM suspension; (**C,D**) eye movement was normal and lagophthalmos was relieved.

**Figure 6:**
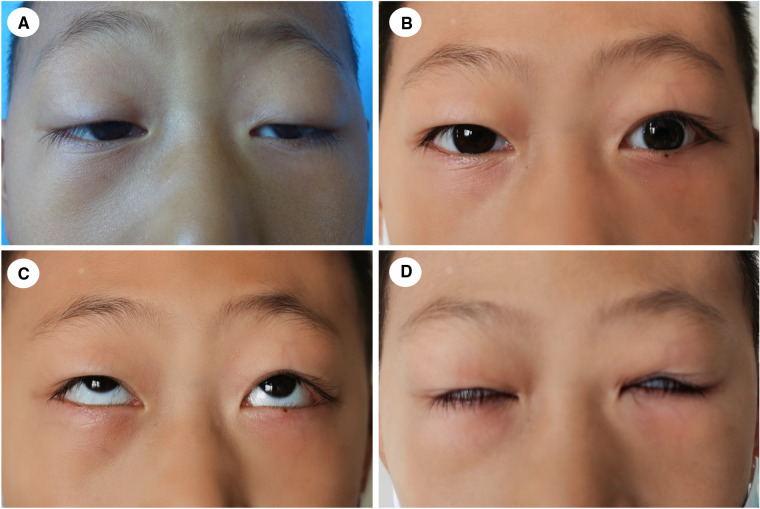
A 9-year-old boy with congenital blepharoptosis OU. (**A**) preoperative appearance; (**B**) twelve months after CFS + LM suspension; (**C,D**) eye movement was normal and lagophthalmos was relieved.

Previous reports on CFS surgery focused on adult patients who could tolerate local anesthesia, and the position between the upper eyelid margin and the upper corneal margin could be observed in the sitting position during the operation to achieve better surgical results. It is unable to make precise designed before the CFS + LM suspension, which was a major challenge for doctors treating pediatric patients; consequently, the upper eyelid margin was adjusted to 0–1 mm from the upper corneal margin during general anesthesia. However, depending on the depth of general anesthesia, the position of the cornea can also change during the operation. Therefore, in addition to referring to the upper corneal margin, we also paid attention to the size of the palpebral fissure, which was set to 8 mm. In this study, good surgical results were achieved after surgery in both group A and group B. The mean MRD1 of group A at the last visit was 3.00 ± 0.69 mm, which approximates the result of 3.10 ± 0.22 (22). The corrective effect was 77.78% at the last visit in group A, which is better than the result of 67.92% in a previous report (23). In view of the long-term outcomes after the operation, there was no significant difference between group A and group B. Therefore, modified CFS suspension under general anesthesia is safe and effective for the correction of severe congenital blepharoptosis in pediatric patients and is worth applying in clinical practice. In Figures 1, 5, 6, we listed three successful cases of congenital blepharoptosis who received CFS + LM suspension.

Moreover, we divided the patients into two groups depending on age to explore whether age affects the outcomes of the surgery. The upper eyelid suspension support system is a complex system composed of muscles, aponeurosis and other tissue. Various components have synergistic or buffer effects. The orbital wall of children develops rapidly, reaching 90% of the adult volume by the age of 5 and, before the age of 7, reaching a state similar to that in adults (24). The eyelid soft tissue also changes rapidly. In our study, there was a statistically significant difference in MRD1 between group A and group B at 6 months after the operation (*P* < 0.05) but not at the last visit (*P* > 0.05). Additionally, there were varying degrees of regression after suspension, especially 3 months after the operation, which was confirmed in a previous report. This result suggested that the third month after the operation was the key period of this treatment.

All patients had early incomplete eyelid closure, which resolved at 1 month after surgery, and the eyelid closure improved over time, which was not bothersome for the patients or their parents. The lower the occurrence of lagophthalmos greater than 2 mm, the lower was the occurrence of exposure keratopathy after surgery. Only two patients presented exposure keratopathy in the follow-up period. Postoperative conjunctival prolapse was found in both group A and group B, which was considered related to the young age of patients and to postoperative conjunctival edema. The edematous conjunctiva fell below the upper edge of the eyelid due to gravity, and conjunctival prolapse recovered as the edema subsided. For severe conjunctival prolapse that could not recover, fixation of the conjunctival fornix with deep sutures was performed. The sutures were removed 10 days later, and the conjunctival prolapse recovered well. Attention should be given to the following points to prevent complications: ① During the operation, it is easy to damage the conjunctiva when separating the Muller's muscle and upper eyelid conjunctiva in the direction of the upper eyelid fornix, leading to postoperative conjunctiva perforation or aggravating postoperative edema. Therefore, the maneuvers should be performed gently to minimize stimulation of the conjunctiva. ② Surgical separation above the fornix may cause varying degrees of conjunctival prolapse or even blepharon prolapse and reversal if the anatomical operation is not skillfully conducted (25). The dissection area above the fornix was not too large, and the CFS was sutured appropriately. Once conjunctival prolapse occurs during the operation, the conjunctiva can be fixed on the aponeurosis of the upper eyelid by 5-0 absorbable sutures before the incision is stitched. If the incision has been sutured, the suture can be ligated on the skin surface under the eyebrow. Early postoperative conjunctival prolapse can be treated under topical anesthesia with instruments to retrieve the conjunctival prolapse, and eye ointment can be administered, along with bandaging of the affected eye. If the prolapse of the conjunctiva cannot recover 1 month after edema revolution by the abovementioned method, the prolapsed conjunctiva needs to be excised surgically. There were fewer postoperative complications, including LAG, exposure keratopathy and conjunctival prolapse, in group B than in group A (*P *< 0.05). Younger patients tended to have unsatisfied compliance due to ocular pain, swelling and other reasons in the first month after surgery.

In conclusion, CFS + LM suspension, a modified CFS-based surgery, is an effective treatment for severe congenital blepharoptosis in pediatric patients with excellent long-term outcomes. Although the upper eyelid position slightly retracts after the operation, it is still safe and feasible for correcting severe congenital blepharoptosis in pediatric patients under general anesthesia. This procedure maintains relatively normal functioning of the levator muscle, which results in better movement of the eyelid, good eyelid closure and fewer complications, which is worthy of further clinical application to cure severe congenital blepharoptosis in pediatric patients.

## Data Availability

The raw data supporting the conclusions of this article will be made available by the authors, without undue reservation.

## References

[1] El ER Elsada MA. Clinical and demographic characteristics of ptosis in children: a national tertiary hospital study. Eur J Ophthalmol. (2013) 23:356–60. 10.5301/ejo.5000239 23483501

[2] Cho BJ Choi YJ Shin MC Yang SW Lee MJ. Prevalence and risk factors of childhood blepharoptosis in Koreans: the Korea National Health and Nutrition Examination Survey. Eye. (2020) 34:1585–91. 10.1038/s41433-019-0697-3 31772383 PMC7608270

[3] Berry-Brincat A Willshaw H. Paediatric blepharoptosis: a 10-year review. Eye. (2009) 23:1554–9. 10.1038/eye.2008.311 18949007

[4] Harvey DJ Iamphongsai S Gosain AK. Unilateral congenital blepharoptosis repair by anterior levator advancement and resection: an educational review. Plast Reconst Surg. (2010) 126:1325–31. 10.1097/PRS.0b013e3181ebe1e9 20885254

[5] Zhou X Zhu M Lv L Jin R Yang J Liu F. Treatment strategy for severe blepharoptosis. J Plast Reconstr Aesthet Surg. (2020) 73:149–55. 10.1016/j.bjps.2019.06.037 31481318

[6] Holmstrom H Santanelli F. Suspension of the eyelid to the check ligament of the superior fornix for congenital blepharoptosis. Scand J Plast Reconstr Surg Hand Surg. (2002) 36:149–56. 10.1080/028443102753718023 12141202

[7] Xing Y Wang X Cao Y Ding X Lin M Li J Modified combined fascia sheath and levator muscle complex suspension with muller muscle preservation on treating severe congenital ptosis. Ann Plast Surg. (2019) 82:39–45. 10.1097/SAP.0000000000001657 30325839

[8] Li Y Wang H Bai P. Changes of ocular surface before and after treatment of blepharoptosis with combined fascial sheath suspension and frontal muscle flap suspension. J Craniofac Surg. (2021) 32:e698–701. 10.1097/SCS.0000000000007693 34172687 PMC8549455

[9] Liu Z Jia X Pang R Wang H Shi J Bai P. Research on the expression of elastin in the conjoint fascial sheath for the correction of severe unilateral congenital blepharoptosis. Bmc Ophthalmol. (2022) 22:256. 10.1186/s12886-022-02469-w 35676638 PMC9175472

[10] Holmstrom H Bernstrom-Lundberg C Oldfors A. Anatomical study of the structures at the roof of the orbit with special reference to the check ligament of the superior fornix. Scand J Plast Reconstr Surg Hand Surg. (2002) 36:157–9. 10.1080/028443102753718032 12141203

[11] Li B Yang J Wu W Chai C Gu Z He Z Anatomical and histological study of the conjoint fascial sheath of the levator and superior rectus for ptosis surgery. Ophthalmic Plast Reconstr Surg. (2020) 36:617–20. 10.1097/IOP.0000000000001660 32251174

[12] Zhou J Chen W Qi Z Jin X. Minimally invasive conjoint fascial sheath suspension for blepharoptosis correction. Aesthetic Plast Surg. (2019) 43:956–63. 10.1007/s00266-019-01382-w 31037324

[13] Lee V Konrad H Bunce C Nelson C Collin JR. Aetiology and surgical treatment of childhood blepharoptosis. Br J Ophthalmol. (2002) 86:1282–6. 10.1136/bjo.86.11.1282 12386090 PMC1771363

[14] Cates CA Tyers AG. Outcomes of anterior levator resection in congenital blepharoptosis. Eye. (2001) 15:770–3. 10.1038/eye.2001.247 11827000

[15] Bernardini FP Cetinkaya A Zambelli A. Treatment of unilateral congenital ptosis: putting the debate to rest. Curr Opin Ophthalmol. (2013) 24:484–7. 10.1097/ICU.0b013e328363861a 23925061

[16] Yoon JS Lee SY. Long-term functional and cosmetic outcomes after frontalis suspension using autogenous fascia lata for pediatric congenital ptosis. Ophthalmology. (2009) 116:1405–14. 10.1016/j.ophtha.2009.01.040 19481809

[17] Bernardini FP Devoto MH Priolo E. Treatment of unilateral congenital ptosis. Ophthalmology. (2007) 114:622–3. 10.1016/j.ophtha.2006.11.017 17324711

[18] Kersten RC Bernardini FP Khouri L Moin M Roumeliotis AA Kulwin DR. Unilateral frontalis sling for the surgical correction of unilateral poor-function ptosis. Ophthalmic Plast Reconstr Surg. (2005) 21:412–6, 416–7. 10.1097/01.iop.0000180068.17344.80 16304515

[19] Nguyen VT Hwang TN Shamie N Chuck RS McCulley TJ. Amyloidosis-associated neurotrophic keratopathy precipitated by overcorrected blepharoptosis. Cornea. (2009) 28:575–6. 10.1097/ICO.0b013e318191bdae 19421037

[20] Lee MJ Oh JY Choung HK Kim NJ Sung MS Khwarg SI. Frontalis sling operation using silicone rod compared with preserved fascia lata for congenital ptosis a three-year follow-up study. Ophthalmology. (2009) 116:123–9. 10.1016/j.ophtha.2008.08.049 19019443

[21] Pan X Wei T Wang X Xu C. Clinical efficacy of conjoint fascial sheath suspension and frontalis muscle suspension in treating moderate or severe congenital ptosis and the effects on ocular surface and refractive status. Exp Ther Med. (2020) 20:3278–84. 10.3892/etm.2020.9053 32855698 PMC7444422

[22] Chen W Liu Z Tian Q Niu H Liu F Wang X Levator resection with suspensory ligament of the superior fornix suspension for correction of pediatric congenital ptosis with poor levator function. Eye. (2016) 30:1490–5. 10.1038/eye.2016.165 27518546 PMC5108019

[23] Sang P Fang M Li X Liu C Xi Q. Treatment of severe ptosis by conjoint fascial sheath Suspension. Biomed Res Int. (2021) 2021:1837458. 10.1155/2021/1837458 34840967 PMC8626196

[24] Farkas LG Posnick JC Hreczko TM Pron GE. Growth patterns in the orbital region: a morphometric study. Cleft Palate Craniofac J. (1992) 29:315–8. 10.1597/1545-1569_1992_029_0315_gpitor_2.3.co_2 1643059

[25] Leelapatranurak K Kim JH Woo KI Kim YD. Lacrimal ductule fistula: a new complication of cosmetic lateral canthoplasty. Aesthetic Plast Surg. (2013) 37:892–5. 10.1007/s00266-013-0181-6 23835599

